# Pathogenesis of Autoimmune Cytopenias in Inborn Errors of Immunity Revealing Novel Therapeutic Targets

**DOI:** 10.3389/fimmu.2022.846660

**Published:** 2022-04-06

**Authors:** Manuela Cortesi, Annarosa Soresina, Laura Dotta, Chiara Gorio, Marco Cattalini, Vassilios Lougaris, Fulvio Porta, Raffaele Badolato

**Affiliations:** Paediatrics Clinic and Institute for Molecular Medicine A. Nocivelli, Department of Clinical and Experimental Sciences, ASST- Spedali Civili of Brescia, University of Brescia, Brescia, Italy

**Keywords:** autoimmune cytopenias, target therapy, genetic disorders, inborn errors of immunity (IEI), gene therapy

## Abstract

Autoimmune diseases are usually associated with environmental triggers and genetic predisposition. However, a few number of autoimmune diseases has a monogenic cause, mostly in children. These diseases may be the expression, isolated or associated with other symptoms, of an underlying inborn error of immunity (IEI). Autoimmune cytopenias (AICs), including immune thrombocytopenic purpura (ITP), autoimmune hemolytic anemia (AIHA), autoimmune neutropenia (AN), and Evans’ syndrome (ES) are common presentations of immunological diseases in the pediatric age, with at least 65% of cases of ES genetically determined. Autoimmune cytopenias in IEI have often a more severe, chronic, and relapsing course. Treatment refractoriness also characterizes autoimmune cytopenia with a monogenic cause, such as IEI. The mechanisms underlying autoimmune cytopenias in IEI include cellular or humoral autoimmunity, immune dysregulation in cases of hemophagocytosis or lymphoproliferation with or without splenic sequestration, bone marrow failure, myelodysplasia, or secondary myelosuppression. Genetic characterization of autoimmune cytopenias is of fundamental importance as an early diagnosis improves the outcome and allows the setting up of a targeted therapy, such as CTLA-4 IgG fusion protein (Abatacept), small molecule inhibitors (JAK-inhibitors), or gene therapy. Currently, gene therapy represents one of the most attractive targeted therapeutic approaches to treat selected inborn errors of immunity. Even in the absence of specific targeted therapies, however, whole exome genetic testing (WES) for children with chronic multilineage cytopenias should be considered as an early diagnostic tool for disease diagnosis and genetic counseling.

## Introduction

Inborn errors of immunity (IEIs), also known as primary immunodeficiencies disorders (PIDD), are a group of more than 450 diseases, most of them with a specific monogenic cause (1, 2).

IEI are inherited disorders of the immune system with a broad spectrum of manifestations, starting with an increased susceptibility to infections, but also often including immune dysregulation with autoimmune disease and hyperinflammation, lymphoproliferation, and malignancy (3). In some patients autoimmune and autoinflammatory manifestations, due to the immune dysregulation, could be the only symptoms of IEI (4). Such cases are categorized as “Disease of immune dysregulation” in the most recent classification of IEI from the International Union of Immunological Societies (IUIS) (1). Other types of IEI include immune dysregulation as part of a broader clinical phenotype (5).

Autoimmune diseases are usually multifactorial, but some monogenic autoimmune diseases have been described in children (6). These diseases can be isolated disorders or can be associated with other manifestations of an underlying IEI. In particular, AICs and inflammatory bowel diseases are frequent manifestations of immunedysregulation (4).

Cytopenia, that is the reduction of one or more mature blood cell types in the peripheral blood, can be the first symptom of many IEI. AICs are caused by immune-mediated destruction of hematopoietic cell lineage (7), include immune thrombocytopenic purpura (ITP), autoimmune hemolytic anemia (AIHA), autoimmune neutropenia (AIN), and Evans syndrome (ES) and are common presentations of IEI in the pediatric age, with at least 65% of cases of ES which are genetically determined (6). ITP and AHIA often present as first symptom in adults too. It has also been described that the relative risk of developing autoimmune cytopenia in a patient with IEI is about 120 fold that of the general population (6) (8). However, cytopenias in patients with IEI are not always caused by autoantibodies (8).

In the last decade, we have observed the identification of a new set of genes and defined a unique class of congenital “immunodeficiency” disorders which are more frequently associated with susceptibility to autoimmunity than infection. These findings have helped to clarify the mechanisms that contribute to the development and maintenance of immune tolerance (9). The possibility to exactly define the molecular diagnosis underlying this immune dysregulation greatly affects the prognosis of these diseases. Genetic characterization, especially in cases of early onset, relapsing or refractory and multilineage autoimmune cytopenias, is of fundamental importance as an early diagnosis improves the outcome and allows the setting up of targeted therapy, or gene therapy, which is currently one of the most attractive targeted therapeutic approaches for IEIs.

Despite targeted therapies are not available for any immunodysregulatory disorder, a genetic diagnosis by Next Generation Sequencing (NGS) or Whole Exome Sequencing (WES) may help to establish an unequivocal diagnosis, allowing genetic counseling, and better define genotype/phenotype correlations. An early and unequivocal diagnosis is also essential to ensure the initiation of life-saving therapies, reducing organ complications, and improving the quality of life (10).

## Autoimmune Cytopenias

AICs are acquired conditions characterized by immune-mediated mature peripheral blood cell destruction (11). The term autoimmune cytopenia includes AIHA, ITP, AIN, and multilineage conditions in ES. AIC are heterogeneous disorders that may be the consequence of many conditions such as infections, malignancy, IEI, or rheumatologic disorders (12).

### Autoimmune Hemolytic Anemia

AIHA is anuncommon disorder caused by autoantibodies directed against self-erythrocyte antigens, leading to premature red cell destruction (13). In particular, AIHA is very rare in infancy and childhood (0.2 per 10/year).In these cases it is associated with immune disorders in about 50% of patients (13). AIHA is classified as warm, wAHIA, (IgG autoantibodies) in 90% of pediatric cases (14), cold (IgM autoantibodies) including cold hemagglutinin disease (CAD) and paroxysmal cold hemoglobinuria) or mixed, depending on the thermal range of the autoantibody.

### Immune Thrombocytopenic Purpura

ITP is an autoimmune disorder that induces a premature platelet destruction. ITP is characterized by autoantibodies against platelet glycoproteins, tipically GPIIb/IIIa and GPIb/IX, but ITP pathogenesis is often more complex (12). T cell abnormalities, for example an excessive polarization toward T-helper cell 1 (Th1), Th0, and Th17 cell types, a direct cytotoxic T cell–mediated destruction, and deficiency of T regulatory cells (Tregs) are described (15). A defect in B regulatory cells, megakaryocyte maturation and survival, and myeloid-derived suppressor cells have all also been demonstrated in ITP (12).

ITP is typically self-limited, but, in some cases, thrombocytopenia may be persistent (lasting between 3 and 12 months) or chronic (lasting more than 12 months) (16).

### Autoimmune Neutropenia

AIN is a benign disease typically with its onset in infants or toddlers. It is caused by autoantibodies directed against a patient's own neutrophils, with subsequent peripheral destruction (17). Spontaneous resolution is frequently observed in most cases within months. Children with AIN rarely suffer from severe or invasive or life-threatening infections despite severe neutropenia (14), because circulating neutrophils, although low in number, can display normal anti-microbial activity and they often increase in number during the acute event (18).

### Evans Syndrome

ES is a rare severe autoimmune disorder that affects two or more cell lineages. Initially described as the combination of ITP and AIHA, the recent definition of ES is an autoimmune disorder that affects two or more blood cell lines, occurring together or developing over time. These cytopenias may include ITP, AIHA, or AIN.

## Management of Patients With Autoimmune Cytopenias

Most children with AHIA, ITP, and AIN have a relatively mild to moderate clinical course of the disease, often requiring only observation and treatment with a varying combination of medications.

According to International Guidelines, Prednisolone, or Prednisone, is recommended as first-line therapy for primary warm AHIA. Rituximab (an anti-CD20 monoclonal antibody, specifically direct at the pathogenic B-cell clone) is not specifically authorized for warm AHIA, but the First International Consensus Group has recommended his use in addition to Prednisone as initial therapy in patients with severe disease (i.e., Hb < 8g/l, Evans syndrome) (19–21). Rituximab, if not added to first line therapy, is generally consider the second line therapy (21). AICs are generally self-limiting diseases, but relapses are not uncommon. Splenectomy is recommended in cases of non-response or relapse after Rituximab (22), with the risk of severe infections, and increased risk of thrombosis.

Third line therapies include Azathioprine, Cyclophosphamide, Cyclosporine, Mycophenolate Mofetil, and Bortezomib, an inhibitor of 26S proteasome (20) (21). In totally refractory forms of AHIA, the combination of Bortezomib and Dexamethasone has shown a possible efficacy (23).

For cold agglutinin disease, monotherapy with Rituximab is considered the first-line approach (19), while glucoroticoid should not be used, given the poor therapeutic response (21, 24).

Intravenous immunoglobulins in a single dose (1g/kg) or a short course of corticosteroids represent the first-line treatment for ITP. If patients do not respond to the first-line therapy, or if continuous therapy is needed, the disorder is called refractory ITP, and second-line therapy is indicated: Rituximab, high-dose dexamethasone, thrombopoietin receptor agonists, splenectomy, or cytotoxic drugs represent second line therapies for refractory ITP (14). Many patients do not responded to any treatments (25).

Patients with AIN rarely need a specific treatment, because they can often responded to bacterial and fungal infection. G-CSF (granulocyte colony-stimulating factor, filgrastim) is recommended only for the minority of patients with serious or recurrent infections (19).

Patients with ES should be promptly investigated for underlying causes of AICs, and in all newly diagnosed children an accurate immunological work-up is required to identify a possible genetic cause that may require specific treatment (26). In fact, ES is often associated with mutations in potentially damaging variants in immune genes (6). The management of ES is based on the use of steroids as first-line therapy, successful in about 80% of cases. Rituximab is consider an effective second-line treatment for children who are resistant, relapse or become steroid-dependent. Children with ES and an underlying diagnosis of autoimmune lymphoproliferative syndrome may be treated with immune suppressants, such as mycophenolate mofetil and sirolimus (26). Splenectomy is generally ineffective in ES (14, 27).

### New Drugs for Treatment of Patients With AICs

Novel treatment approaches are actively being developed for the treatment of AICs.

Among the new drugs, B cell directed therapies for example Ofatumumab (anti-CD20), Alemtuzumab (anti-CD52), and Daratumumab (anti-CD38) have been used with good results in case reports for the treatment of secondary AIHAs (28).

Fostamatinib is an inhibitor of spleen tyrosine kinase (SYK) that reduces macrophage-mediated clearance of RBCs and platelets. Syk plays a central role in FcγR-mediated signal transduction, and for this reason it has been considered a target for inhibition in different autoimmune and malignant conditions (29). The blockage of FcγR signaling through the inhibition of Syk ameliorate platelet destruction, and Fostamatinib is now FDA-approved for the treatment of thrombocytopenia in adult with chronic ITP an insufficient response to a previous treatment and is now in Phase 3 studies in wAIHA (20, 30). Orilanolimab, Rozanolixizumab, and Nipocalimab are monoclonal antibodies that bind and inhibit the neonatal Fc receptor (FcRn). The blockage of FcRn induces an increased clearance of IgG including that of pathogenic IgG autoantibodies (31). Rozanolixizumab has already shown efficacy at increasing platelet counts in patients with persistent/chronic primary ITP (32), while the safety and efficacy of Rozanolixizumab and of the other inhibitors of the FcRn for AHIA are under investigation (30).

Ibrutinib is a Bruton tyrosin kinase (BTK) inhibitor currently used or under investigation in several lymphoproliferative disorders (30). BTK inhibition has a significant effect on both lymphocytes and macrophages. His efficacy is reported in secondary AIHA, while a trial with another BTK inhibitor is currently underway for the treatment of ITP (20).

Belimumab is a human monoclonal antibody that inhibits the binding of soluble B lymphocyte stimulator (BLyS, also known as B-cell activating factor, BAFF) to B cells. Belimumab inhibits the survival of B cells (including autoreactive B cells) and reduces the differentiation of B cells into Ig-producing plasma cells. It is currently approved for the treatment of non-renal systemic lupus erythematosus (33), but it might also be a further solution for refractory AIHA patients (34).

### Follow Up of Patients With AICs

In general, if a patient does not responded to first-line therapy, a diagnostic reevaluation should be considered, focusing on any previously overlooked cause of secondary AICs. In these subjects, the disease can be characterized by recurrent, chronic or refractory cytopenias affecting more than one blood lineage (concurrently or sequentially) or be associated with adenopathy and/or hepatomegaly and/or splenomegaly or concurrent severe infections (16). In these cases, a further diagnostic evaluation is needed for the identification of the underlying disorder and, potentially, of novel treatment options.

**Figure 1:**
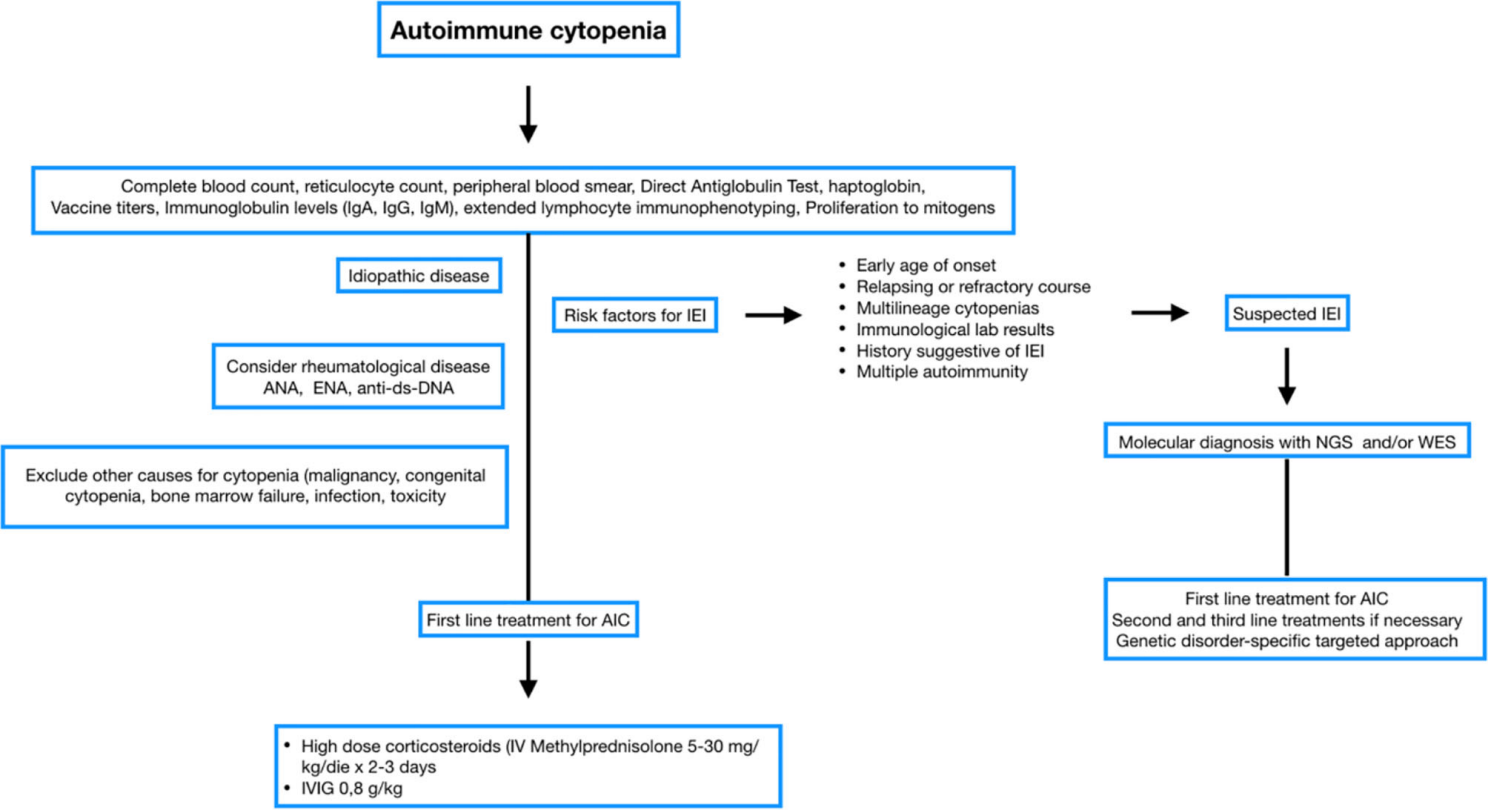
Flow chart Immune cytopenia management. Blood and immunological test at onset. Genetic studies for bilinear cytopenia and relapses.

Evaluation of anti-vaccine antibody response, immunoglobulin levels (IgG, IgA, IgM), extended immunophenotype and proliferation test in response to mitogens, in conjunction with molecular tools for IEI diagnosis are essential for identifying a previously undiagnosed IEI (7, 35). Immunological investigations are always recommended, especially in the case of positive family history for IEI or immunedisregulation, very early onset and relapsing or refractory (11) (**Figure 1**).

Patients with an underlying IEI may often required more second-and third-line immunomodulating therapies and may benefit from a specific target therapy (36) (35).

## Pathogenesis of Autoimmune Complications in IEI

Autoimmunity, defined as the breakdown of immune tolerance to self-antigens, has different pathophysiologic pathways (37). In particular, autoimmune cytopenias may be caused by cellular or humoral immune responses, and often the underlying pathogenetic mechanism involves an immune system defect in which T lymphocytes do not adequately control the proliferation of autoreactive B lymphocites clones (38). Indeed, central and peripheral tolerance usually allows the elimination of lymphocytes reactive to self-antigens, for example through the daily destruction of self-reactive B lymphocytes produced in the bone marrow, avoiding autoimmune diseases (39).

Other possible causes of cytopenia in IEI patients are immune dysregulation (in form of hemophagocytosis or lymphoproliferation with or without splenic sequestration), bone marrow failure and myelodysplasia, and secondary myelosuppression (8).

### Autoimmune Cytopenias Associated With Defect of T Cell Immunity

Central tolerance is one of the function of the thymus, consisting in the elimination of self-reactive T lymphocytes through negative selection, also known as clonal deletion (40). A breakdown of this central tolerance leads to autoimmunity. *Aire* is a gene expressed in thymic medullary epithelial cells, and it mediates the ectopic induction and presentation of many tissue-specific antigens to maturing T cells (41). This antigen presentation promotes the negative selection of autoreactive thymocytes, as well as self-tolerance. If aire is nonfunctional or absent, autoreactive T cells can escape clonal deletion and may later cause autoimmune disease (42). In addition, aire controls immune tolerance by the stimulation of a population of FOXP3-positive T regulatory cells (Tregs) in the thymus that can suppress autoreactive cells, thus influencing peripheral tolerance (43).

Mutations affecting this gene cause autoimmune polyendocrinopathy, candidiasis, and ectodermal dysplasia (APECED), also called autoimmune polyendocrinopathy syndrome type 1 (APS-1).

Levels of aire are also decreased in patients with RAG deficiency and Omenn syndrome thus contributing to the escape and peripheral expansion of autoreactive T cells (44, 45). A defect in peripheral tolerance is also implicated in the development of autoimmunity (46) that coexists with a marked immune impairment.

The thymic aplasia/hypoplasia characterizes DiGeorge syndrome (DGS) resulting in a decreased expression of aire, which may contribute to the autoimmunity often presents in DGS patients (46).

Impaired negative selection of autoreactive T cells as in APECED, RAG deficiency and DGS causes different autoimmune features, including AIC (44).

Thymocyte escape of central deletion can also occur because of abnormalities of TCR signaling, with subsequent defective activation-induced cell death (AICD) as in ORA1 and STIM1 deficiency, which are both characterized by immunodeficiency and autoimmunity manifestations associated with AIC (47).

Many self-reactive cells escape from the central tolerance mechanisms, but other mechanisms operating in the periphery can delete autoreactive lymphocytes.

Apoptosis, the process of programmed cell death, plays a central role in the destruction of autoreactive T and B cells at central and peripheral tolerance checkpoints. Pathogenic variants in genes involved in the apoptotic pathway (fas, fasl, casp10) result in the uncontrolled proliferation and accumulation of autoreactive (TCRalphabeta-/CD4+/CD8+ double-negative) T cells. The death receptor FAS, his ligand FASL, and the other genes of the FAS/FASL pathway (fadd, casp8, casp10) play a central role in apoptosis both in central and peripheral tolerance. Pathogenic variants in these genes cause autoimmune lymphoproliferative syndrome (ALPS), a primary immunodeficiency characterized by autoimmune disorders with AIC, splenomegaly, lymphadenopathy, and risk of lymphoma (39).

Peripheral tolerance is controlled by a subset of CD4+ CD25+ T cells (Regulatory T cells, Tregs). The development and function of T reg are regulated by Forkhead box protein P3 (foxp3), which affects T regs functional activity. Variants in foxp3 and related components cause a reduction in the number and diversity of Tregs, with severe consequences in the maintenance of peripheral tolerance leading to immune dysregulation, polyendocrinopathy, enteropathy, X-linked (IPEX) with a very early onset (47, 48).

Development and functioning of T reg cells depend on IL-2 mediated signaling, and defects in IL-2 signaling, involving STAT5b and CD25, lead to IPEX- like syndromes, with similar autoimmune and autoinflammatory symptoms.

The adaptative immune response is strictly controlled by positive and negative regulators.

The positive signal is induced by the T cell receptor (TCR) on naïve T cells through the recognition of the antigen presented by the MHC complex expressed on the antigen-presenting cells (APCs). The second signal is the formation of an immunological synapse (IS), through the binding of the costimulatory T- cell receptor CD28 on the T-cell surface to B7 molecules (CD80 and CD86) on the APCs (i.e dendritic cells, macrophages and B cells). The CD28/B7 interaction is essential for IS creation, as it is required to ensure a complete T-cell activation (49). Cytoskeletal proteins are essential for many cellular functions, including the IS between T cells and APCs, and the regulation of lymphocyte proliferation. Variants in *was* are responsible of Wiskott-Aldrich syndrome. *Was* gene econdes WAS protein, an actin-nucleation promoting factor expressed in hematopoietic stem cells. WAS is characterized by the triad of thrombocytopenia with small-size platelets, eczema, and lymphopenia, mainly affecting T cells. The absent function of was is reflected by ineffective T cell proliferation and function, reduced Treg activity (with normal number of Treg), and hyperproliferation of B cells, which subsequent production of autoantibodies (50). Autoimmune manifestation are common, with AIHA, autoimmune neutropenia, peripheral vasculitis, and arthritis considered the most common.

Tregs act as negative regulators of adapative immune response. Defective Tregs development and function associated with autoimmune and lymphoproliferative disease are described in pathogenic variants of cytotoxic T-lymphocyte-associated protein 4 (ctla-4), lipopolysaccharide responsive beige-like anchor protein (lrba), and differentially expressed in FDCP6 homolog (def6) (47).

Ctla-4 is an essential negative immune checkpoint constitutively expressed in Treg cells and it is an inhibitor of T cell activity. Ctla-4 impairment is also observed in biallelic mutations of lrba and def6. Therefore, patients with CTLA-4 insufficiency, LRBA, and DEF6 deficiency have a similar clinical phenotype due to a defective suppressive activity of Tregs, as all three diseases present with reduced expression or defective function of CTLA-4 (49).

CTLA-4 is the CD28 homologue protein that “switches off” the T-cell-dependent response after pathogen elimination. CTLA-4 is mostly intracellularly stored in naïve T cells or constitutively expressed, and up-regulated under stimulus on the surface of Tregs. Upon TCR-dependent activation, when naïve T cells activation through the TCR, CTLA-4-is mobilized to the cell surface, forming homodimers that outcompete CD28 and bind to B7 molecules with higher affinity and avidity, activating cell-intrinsic and cell-extrinsic inhibitory mechanisms.

Lrba and def*6* essentially control CTLA-4 recycle. Thereby, pathogenic variants in these genes lead to increased CTLA-4 lysosomal degradation and decreased CTLA-4 recycling.

The reduction of CTLA-4 on the T-cell surface is the common hallmark of ctla*-4* insufficiency, lrba, and def*6* deficiency, and induces immune homeostasis disruption due to the prolonged T-cell activation and migration, with subsequent multiorgan lymphocytic infiltration and a breakdown of the peripheral immune tolerance. A higher circulation of autoreactive lymphocytes may explain the development of autoimmunity, including AIC (51). Autoimmunity (AIC, enteropathy) is often associated with recurrent and/or severe infections, hypogammaglobulinemia, and lymphoproliferation.

Signal Transducers and Activator of Transcription (STAT) molecules play a central role in different signaling pathways activated by several cytokines and are responsible for the transcription of genes essential in the immune and inflammatory response (52). STAT1 signaling is activated by multiple cytokines including IFN-α and IFN-gamma, while STAT3 mediates responses to IL-6. The presence of Janus kinase (JAK) molecules is required for STAT1 and STAT3 phosphorylation. Impaired or enhanced function of the JAK/STAT-dependent molecular pathways results in immune dysregulation and susceptibility to infections.

In stat*1* gain of function (GOF), due to reduced TH17 cells proliferation, patients present with an increased susceptibility to different bacterial and fungal infections, resulting in chronic mucocutaneous candidiasis and pulmonary infections (53). Autoimmunity and immune dysregulation are reported in 30% of patients with *stat1* GOF mutations, mainly represented by endocrinopathies, blood cytopenias, and enteropathy (54, 55).

*Stat3* GOF mutations result in a combined immune defect (56), featured by an increased risk of severe infections, and with a high incidence of autoimmunity (cytopenia, enteropathy, endocrinopathy, arthritis), lymphoproliferation, hypogammaglobulinemia and hepato-splenomegaly.

### Autoimmune Cytopenias Associated With Defects of Humoral Immunity

Among humoral defects, autoimmunity is a common feature in patients with Common Variable Immunodeficiency (CVID, including those with taci defect, baff-r defect, icos, *NF-kB1* and *NF-kB2* deficiency), selective IgA Deficiency (sIgAD), and hyper-IgM syndrome (HIGM). Autoimmune manifestations, such as arthritis, type 1 diabetes, intestinal bowel diseases, and AIHA are reported in 15% of patients with X-linked agammaglobulinemia (XLA) (48).

CVID is characterized by hypogammaglobulinemia (low IgG and IgA, with or without low IgM levels) with poor antibody response to vaccines or low switched-memory B cells, according to ESID criteria (57). Non-infectious manifestations, including autoimmune, lymphoproliferative disorders, and malignant diseases are present in about 30-50% of CVID patients (58). Autoimmune diseases are described in 20–30% of CVID patients; ITP, AIHA, and ES are the most frequent and can constitute the first manifestation of the immune defect, even in the absence of infection history. Development of autoimmunity can be related to several mechanisms including an altered reaction of the germinal center resulting in abnormal class switch, or uncontrolled B cell proliferation (59). Other mechanisms may include the defective suppressive function of B-regs on activated T cells (60); or hyperactivated T cell phenotypes (61).

CVID is mainly a polygenic disease, but a monogenic cause can be identified in about 15-30% of cases (62). Defects in the common receptor of *taci*, of the B cell-activating factor *(baff)* and *april* (a proliferation-inducing ligand) encoded by the *tnfrsf13b* gene, have been linked to CVID. CVID patients with CVID-associated variants of *tnfrsf13b* especially if heterozygous, have a higher risk for autoimmune complications and lymphoid hyperplasia potentially due to lack of normal mechanisms required to establish tolerance. Pathogenic variants in *baff-r*, impairing B-cell maturation, and in *NF-kB1* and *NF-kB2*, transcription factors essential for B-cell maturation, survival, differentiation, class switching, and self-tolerance, are also associated with autoimmunity.

The HyperIgM (HIGM) syndromes include a group of IEIs caused by defective class-switch recombination, resulting in low levels of IgG, IgA, and IgE with normal or increased levels of IgM (63). HIGM syndromes are heterogeneous diseases, with X-linked, autosomal recessive, and autosomal dominant inheritance. Variants in the gene encoding CD40 ligand (CD40L), a protein expressed on activated T cells, are the most common cause of X-linked HIGM. Autosomal recessive forms of HIGM are associated to biallelic mutations in *aid* and *ung.*

Often, but not always, HIGM syndromes are characterized by the association of immunodeficiency and autoimmune diseases, depending on the genetic background. In the X-linked form, for example, self-reactive B-cells cannot be eliminated due to the defective CD40-CD40L mediated interaction, that is also responsible for the reduction of Tregs, while in the recessive form, AID deficiency could hesitate in impaired regulation of self-reactive B cells (59).

Activated phosphoinositide 3-kinase delta syndrome (APDS) is caused by heterozygous gain of function mutations in *pi3kδ* catalytic p110δ (*pik3cd*) or regulatory p85α (*pik3r1*) subunits leading to APDS1 and APDS2, respectively (64). One of the major downstream effectors of PI3K is mTOR, which plays a central role for cell growth and survival and in TH1 and TFH cell differentiation (65). APDS is characterized by the proliferation of effector cells, while naïve, and central memory T-cell subsets remain quiescent, with CD8 senescence, and imbalance between immature and mature cells in the T compartment, likely contributing to autoimmunity, lymphoproliferation, and immunodeficiency seen in this syndrome (55). AICs are the most frequently reported (76.2%) autoimmune manifestations (64).

## Targeted Therapy in Immune Dysregulation Disorders of AICs

The main focus of IEI management is treating their clinical manifestations, and avoiding complications using antibiotics, immunoglobulin replacement, corticosteroids, and immunosuppressive agents, and it mainly depends on the underlying IEI.

**Figure 2:**
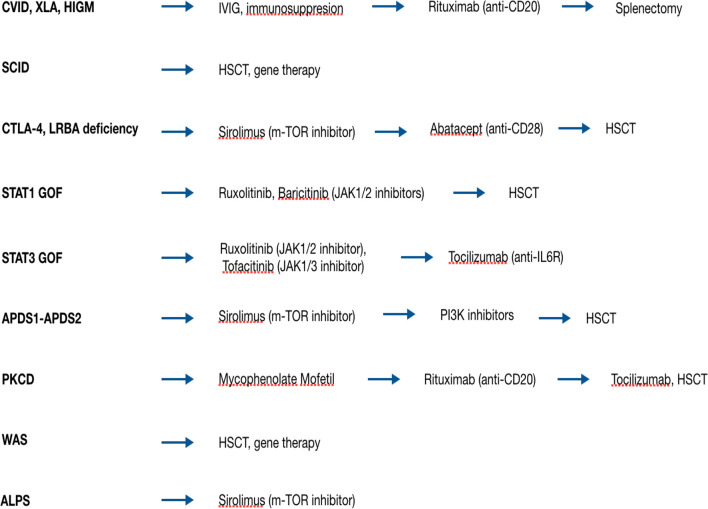
Therapeutic strategies for AICs in patients with IEIs.

The broad availability of whole exome and whole genome sequencing analysis has made possible the discovery of an increasing number of genes, responsible for new genetic disorders. The capability to exactly identify the molecular basis of IEI has made possible to target the genetic defect with therapeutic agents. These new drugs can replace or can be combined with traditional immunosuppressant agents, both in the treatment of AIC and treat the other clinical manifestation of IEIs. Target agents act modulating the activity of intracellular pathways, whose function is either decreased or increased due to a certain gene defect (66) (**Figure 2**).

### Autoimmune Lymphoproliferative Syndrome

ALPS is characterized by hyperactivation of PI3K/Akt/mTOR signaling pathway. Sirolimus, an mTOR inhibitor, is used with great success in children with ALPS, reducing lymphoproliferation. The treatment is described as particularly effective in ALPS patients who have autoimmune cytopenias (67).

### CTLA4 Haploinsufficiency

Along with symptomatic therapies (anti-infective and anti-inflammatory treatment, first-line immunosuppressive treatment against autoimmune cytopenias and inflammatory parenchymal lung disease), many patients benefit from rapamycin (mTOR) inhibitors, and soluble CTLA-4-Ig (Abatacept, Belatacept), functioning *in vivo* as inhibitors of T-cell activation, mimicking CTLA4 function (68). In particular, the efficacy of Abatacept in auto-immune cytopenias caused by CTLA-4 haploinsufficiency has been well characterized (69). It is important to underline that the possibility of viral infection/reactivation during the treatment with Abatacept or Belatacept may be a limitation for the use of these drugs. For this reason, hematopoietic stem cells transplantation may be considered a possible definitive therapy (39).

### LRBA Deficiency

The phenotypic overlap between CTLA4 and LRBA deficient subjects and their common cellular pathway establish a rational basis for the treatment with Abatacept. Abatacept mimics cellular CTLA4 function, rendered missing by LRBA deficiency, and negative regulate the immune responses by blockading or capturing CD80/86 molecules (70).

However, as in CTLA4 haploinsufficiency, the only curative therapy is HSCT: earlier studies showed that most patients with LRBA deficiency who have undergone HSCT achieve complete immune reconstitution (71).

### IEI Associated With STAT1 or STAT3 Gain of Function Mutations

Autoimmune manifestations (blood cytopenias, juvenile diabetes, hypothyroidism) are common in STAT1 GOF patients and they are often refractory to conventional treatment and also to the treatment with multiple immunosuppressants (72).

Although HSCT is the only curative treatment, the molecular understanding of *JAK-STAT* pathway has allowed the use of target pharmacologic inhibitors in patients with STAT1 GOF. Currently 5 different JAK inhibitors are available: Tofacitinib (JAK1 and JAK3 inhibitor), Ruxolitinib and Baricitinib (JAK1 and JAK2 inhibitors), Filgotinib (selective JAK1 inhibitor), and Decernotinib (selective JAK3 inhibitor) (66).

Jakinibs are also used for the treatment of STAT3 GOF patients, as an alternative to the anti-IL6 receptor (IL6R) mAb Tocilizumab. Indeed, IL6 and other cytokines, such as type I, II, and III interferons, IL-10, and IL-21, activate STAT3-mediated intracellular signaling. The combination of IL6 blockade and Jakinib therapy has proved to be an effective strategy for the treatment of immune dysregulation in STAT3 GOF patients (72).

### Activated Phospoinositide 3-Kinase Delta Syndrome

Standard treatments for APDS include antimicrobial prophylaxis and immunoglobulin replacement as anti-infective prevention (73). Different immunosuppressive regimens (steroids, Rituximab, and calcineurin and rapamycin inhibitors) in different combinations can be used to control the symptoms of lymphoproliferation and autoimmunity (74). Most of APDS clinical symptoms were improved after HSCT, but an elevated rate of post-HSCT viral reactivation and engraftment failure have been reported (75, 76). Selective PI3K delta inhibitors constitute a targeted therapy based on the molecular mechanisms of APDS. Drugs such as leniolisib and nemiralisib have been successfully used, resulting in the reduction of lymphoproliferation, and improvement of clinical manifestations, including autoimmune cytopenia episodes (77, 78).

### New Treatment Strategies of AICs in Patients With IEI

Several trials with new drugs (B-cell directed monoclonal antibodies; B-cell receptor inhibitors; IgG-mediated phagocytosis inhibitors) are ongoing or being planned. Given their mechanism of action, a potential use in AIC with underlying immunodeficiency may be envisioned. However, no specific information regarding IEI is currently available.

### Treatment of IEI Patients With Hematopoietic Stem Cel Transplantation and Gene Therapy

Allogeneic hematopoietic stem cell transplantation constitutes the only life-saving and curative treatment for some severe IEIs (ie: SCID) who currently do not benefit from any target therapy.

As mentioned above, also non-SCID patients may benefit of HSCT, requiring a complete conditioning regim to achieve engraftment.

Other management options, for example enzyme replacement, and gene therapy may provide an alternative approach to HSCT in specific IEIs.

Gene therapy is a relatively new approach to IEIs, based on the infusion of autologous hematopoietic stem cell (HSCs) transplantation to deliver stem cells with added or edited versions of the gene of interest (79). Gene therapies with retroviral and lentiviral vectors are being developed for different IEIs and may be find an application in many immunedeficiencies.

## Conclusion

The ability to identify the correct molecular diagnosis in IEI has a direct impact on prognosis and it has opened the pathway for the use of novel target therapies which are capable to correct abnormal functioning of the immune response.

These therapies have shown to be particularly effective in patients with hyperinflammation and immune dysregulation, but access to this new family of drugs requires precise identification of the genetic basis of the disease. Additional studies are needed to evaluate whether the use of precision therapies can optimize disease management in children with autoimmune cytopenia.

## Author Contributions

MCo wrote the initial manuscript. VL, MCa, CG, LD, AS, and FP reviewed and revised the manuscript. RB critically reviewed the manuscript for important intellectual content and contributed to the review. All authors approved the final manuscript as submitted and agree to be accountable for all aspects of the work.

## Conflict of Interest

The authors declare that the research was conducted in the absence of any commercial or financial relationships that could be construed as a potential conflict of interest.

## Publisher’s Note

All claims expressed in this article are solely those of the authors and do not necessarily represent those of their affiliated organizations, or those of the publisher, the editors and the reviewers. Any product that may be evaluated in this article, or claim that may be made by its manufacturer, is not guaranteed or endorsed by the publisher.
